# Epidemiology of dengue hemorrhagic fever in Indonesia: analysis of five decades data from the National Disease Surveillance

**DOI:** 10.1186/s13104-019-4379-9

**Published:** 2019-06-20

**Authors:** Harapan Harapan, Alice Michie, Mudatsir Mudatsir, R. Tedjo Sasmono, Allison Imrie

**Affiliations:** 1 Medical Research Unit, School of Medicine, Universitas Syiah Kuala, Banda Aceh, 23111 Indonesia; 2 School of Biomedical Sciences, University of Western Australia, Nedlands, WA 6009 Australia; 3 Department of Microbiology, School of Medicine, Universitas Syiah Kuala, Banda Aceh, 23111 Indonesia; 4 Eijkman Institute for Molecular Biology, Jakarta, 10430 Indonesia

**Keywords:** Dengue hemorrhagic fever, Epidemiology, Incidence rate, Mortality rate, Indonesia

## Abstract

**Objective:**

To provide a national incidence rate and case fatality rate of dengue hemorrhagic fever in Indonesia through an analysis of the National Disease Surveillance database from the Directorate General of Disease Prevention and Control of Ministry of Health.

**Results:**

Available data has indicated an increasing trend of dengue hemorrhagic fever incidence in Indonesia over the past 50 years. Incidence rates appear to be cyclic, peaking approximately every 6–8 years. In contrast, the case fatality rate has decreased approximately by half each decade, since 1980. Java Island contributed the highest average number of dengue hemorrhagic fever cases each year. In recent years, Bali and Borneo (Kalimantan) have had the highest incidence while Papua Island, the easternmost region of the Indonesian archipelago, has had the lowest incidence.

## Introduction

Dengue, caused by infection with any of the four dengue virus (DENV) serotypes (1–4), is the most important mosquito-borne viral disease in humans and is of major public health concern [1, 2]. The clinical manifestations of DENV infection range from asymptomatic infection or a mild flu-like syndrome, also known as dengue fever (DF), to the more severe and life-threatening forms, dengue hemorrhagic fever (DHF) and dengue shock syndrome (DSS) [3]. It has been estimated that 390 million DENV infections occur annually, worldwide [4], of which 50–200 million are apparent cases (symptomatic infections, including those that are undetected by reporting systems) [4, 5]. Data from 76 countries have indicated a substantial increase in dengue incidence, where the number of apparent cases has more than doubled every decade between 1990 and 2013, with the highest incidence of infection being reported in Asian countries [5].

Indonesia, a transcontinental unitary sovereign state located in Southeast Asia, is a tropical country where both main mosquito vector species of DENV, *Aedes aegypti* and *Ae. albopictus*, are endemic almost in all regions [6]. Dengue prevention and control programs have been in place on a national scale by the Ministry of Health (MoH) of Indonesia through the Directorate General for Communicable Diseases Control since 1968 [7]. The programs include the implementation of peri-focal adult spraying, mass larviciding, and disease control education to the community. Despite the efforts of these control programs, dengue has expanded in both incidence and geographical range over the years and has become hyperendemic with multiple co-circulating DENV serotypes, nationwide. Several major dengue outbreaks have been reported in the country [8–15].

Since the first dengue reports in Jakarta and Surabaya in 1968, the epidemiology of dengue in Indonesia has changed [16, 17]. These changes have included more irregular DHF outbreaks, with a high inter-epidemic background [16], increasing mean age of DHF cases, increasing annual incidence rate (IR), and decreasing case fatality rate (CFR) [16, 17]. Our group has assessed several aspects of dengue in Indonesia, including seroprevalence [18, 19], molecular epidemiology among locals [15, 20–25] or travellers returning from Indonesia [26], as well as public health aspects [27–32]. Currently, we also have provided a comprehensive national picture of DENV circulating in Indonesia [33]. Missing from the literature is an update on the burden of dengue (i.e. incidence and mortality of the disease). Previously, national IR and CFR of DHF have been reported [16, 17] but there has been no update since 2014. The aim of this study was to provide an update of the national epidemiology of DHF in Indonesia over five decades using the National Disease Surveillance database from the MoH of Indonesia.

## Main text

### Methods

#### Data source

Dengue has been a notifiable disease in Indonesia since 1968 and is reported continuously into the National Disease Surveillance run by the Directorate General of Disease Prevention and Control of the Indonesian MoH. Reporting of DHF by Community Health Centres (*Puskesmas*) and public or private hospitals to district health authorities is mandatory in Indonesia within 72 h of diagnosis. To provide a comprehensive national IR and CFR of DHF in Indonesia overtime, we analysed the surveillance database from 1968 to 2017.

#### Case definition and criteria

Details of DHF case definition and case ascertainment used in this surveillance have been published elsewhere [17]. In brief, since its inception in 1968, the surveillance system has used the World Health Organization (WHO) dengue classification system, which classified symptomatic dengue into DF and DHF [34]. Individuals with mild symptomatic DF and those not presenting to healthcare facilities were not captured by the surveillance system [17]. Although the dengue classification system and criteria have changed since 1968, definitions and criteria of DHF used in this surveillance system have remained stable over the entire time period [17].

Dengue hemorrhagic fever was defined as having at least the first two of the following four clinical manifestations: (a) sudden onset acute fever of 2 to 7 days duration; (b) spontaneous hemorrhagic manifestations or a positive Tourniquet test; (c) hepatomegaly; and (d) circulatory failure, in combination with haematological criteria of thrombocytopenia (≤ 100,000 cells/mm^3^) and an increased haematocrit over 20% [17]. Suspected DHF cases based on those criteria were further assessed in which DHF cases were classified into probable and confirmed cases. A probable case was defined as clinically suspect patients supported by positive dengue serology (positive anti-DENV IgM in acute or convalescent serum sample and/or a fourfold increase in IgG between the acute and the convalescent samples) or when a suspected DHF patient was linked at the same location and time to other confirmed DHF cases. A confirmed case was defined as a case with laboratory confirmation through DENV isolation, or detection of viral antigen or RNA in serum. This classification has continually been used nationwide by hospitals and Community Health Centres in Indonesia [17]. All probable or confirmed DHF cases were reported and included in the surveillance system.

#### Data collection process and synthesis

The number of cases and deaths associated with DHF, recorded from between 1968 and 2017, were retrieved from that National Dengue Surveillance registry. The annual IRs of DHF were determined by dividing the number of new DHF cases identified, by the size of the population at risk for the correspondent year (the total Indonesian population). The annual Indonesian population size used in the analysis was based on population number used by the MoH and reported in its annual report (Indonesian Health Profile). For most years, the MoH used the population size derived from the official database of Indonesian Central Bureau of Statistics. The IRs were expressed as per 100,000 person-years. The CFRs were calculated as the number of deaths associated with DHF divided by the number of DHF cases, expressed as a percentage (%).

To determine the national geographical distribution of DHF cases in Indonesia, geographical mapping of IRs and CFRs of each province, from 2011 to 2016, were created using ArcGIS [35]. The provincial IRs were expressed as the number of case per 100,000 population, while provincial CFRs were expressed as a percentage (%).

### Results

**Fig. 1:**
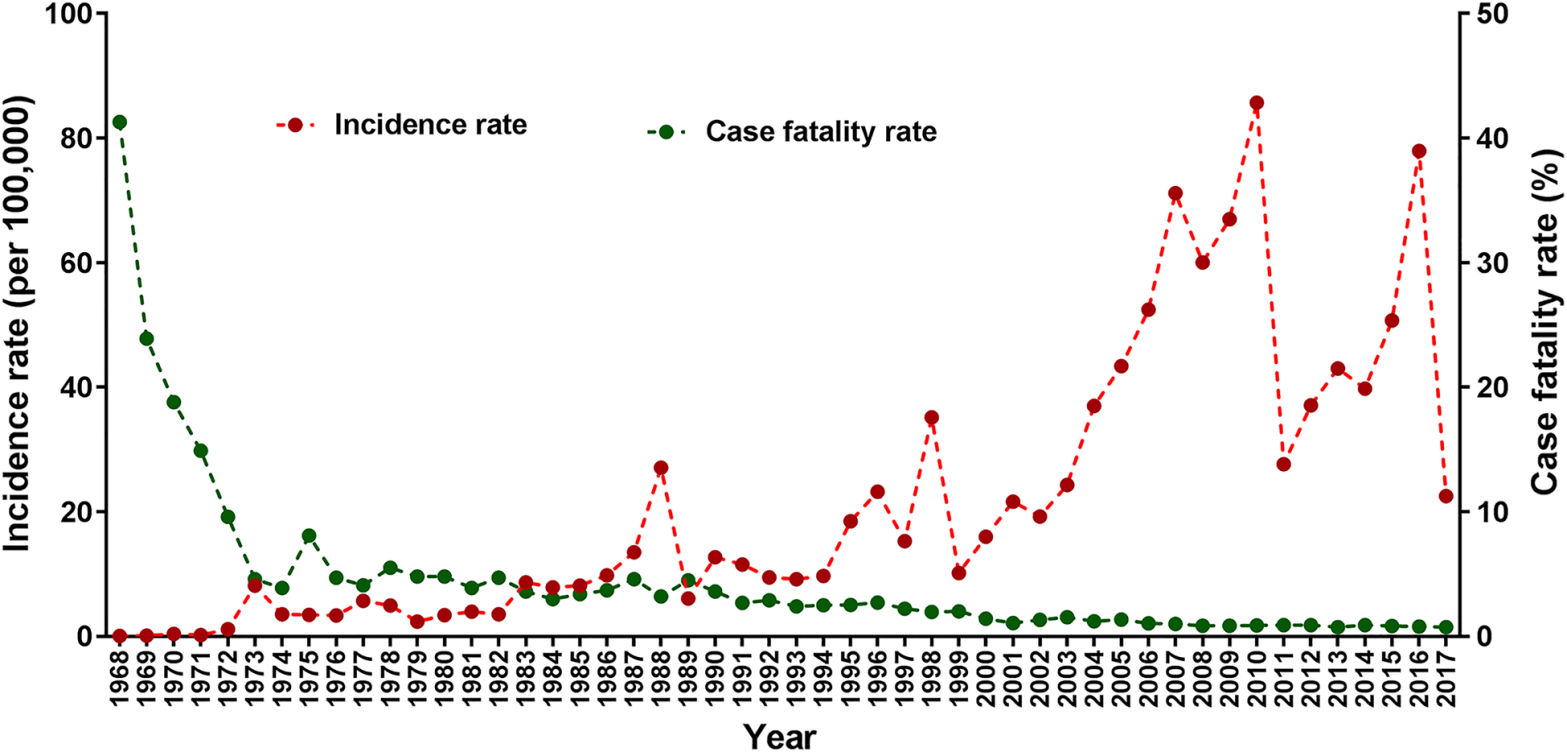
Incidence rate (per 100,000 person-years) and case fatality rate (%) of dengue hemorrhagic fever in Indonesia from 1968 to 2017

Over the 50-year period, there was a sharp increase in the annual IR of DHF in Indonesia, from just 0.05 cases per 100,000 person-years in 1968 to 77.96 cases per 100,000 person-years in 2016 (Fig. 1). The IRs of DHF have had a cyclic pattern, with peaks occurring approximately every 6–8 years. Incidence peaks occurred in 1973, 1988, 1998, 2009, and 2016. In 2017, there were 59,047 and 444 of DHF cases and DHF-associated deaths in Indonesia with 22.55 per 100,000 person-years and 0.75% of IR and CFR, respectively.

Although the annual IR of DHF has increased significantly over the last five decades, the annual CFR has decreased over time. In the late 1960s, the CFR was estimated to be more than 20% of those infected, which subsequently declined approximately by half each decade since 1980. As of 2016, the CFR of DHF was just 0.79% (Fig. 1).

**Fig. 2:**
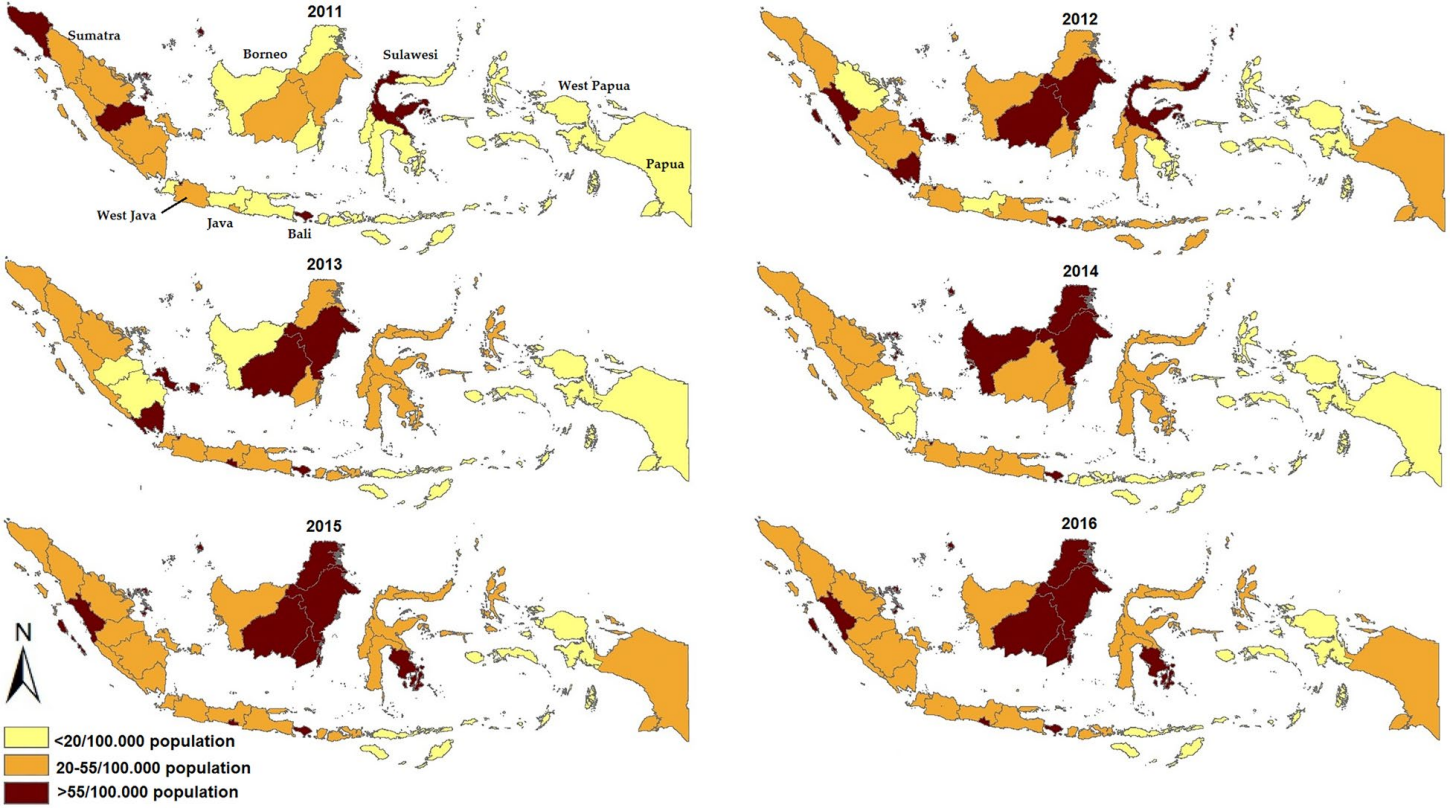
Geographical mapping the provincial incidence rate of dengue hemorrhagic fever (per 100,000 population) in Indonesia from 2011 to 2016

**Fig. 3:**
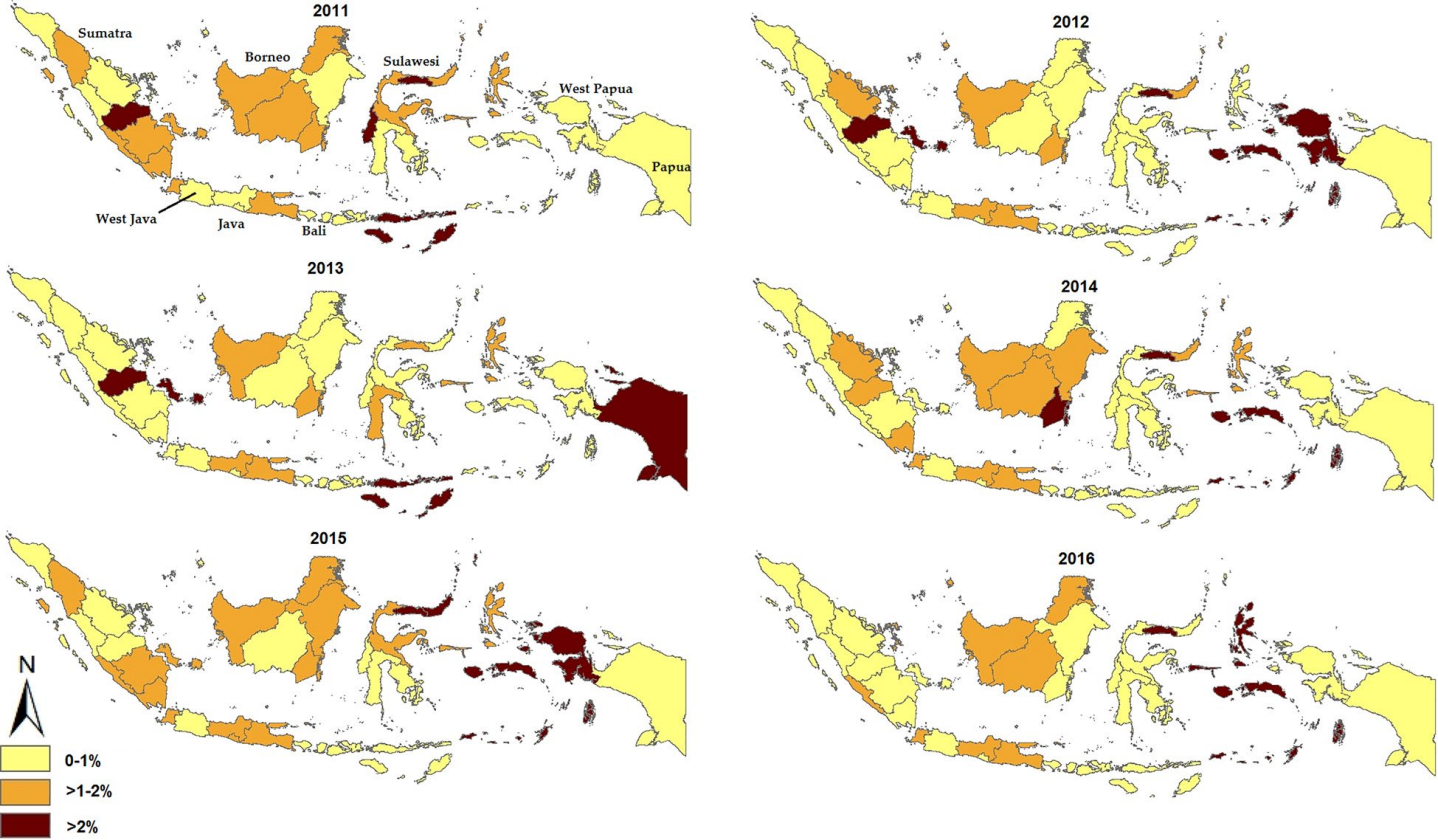
Geographical mapping of the provincial case fatality rate of dengue hemorrhagic fever (%) in Indonesia from 2011 to 2016

Based on provincial geographical mapping of IRs between 2011 and 2016, West Java contributed the highest average number of DHF cases each year (Fig. 2). Bali has reported the highest IR since 2011, ranging from 65.90 per 100,000 population in 2012 to 484.02 per 100,000 population in 2016 (Fig. 2). Interestingly, while Bali has reported the highest dengue incidence annually, the CFR has consistently been less than 1% of those infected (Fig. 3). In contrast, in several provinces where dengue is not endemic such as Papua and West Papua (both provinces formerly known as Irian Jaya), those regions have experienced dengue outbreaks associated with a high CFR, such as the epidemics of 2012 and 2015 in West Papua and the 2013 Papua outbreak (Fig. 3). Overall, in recent years, it is clear that Bali and Borneo (Kalimantan) have had the highest IR of DHF while West Papua has had the lowest IR in Indonesia.

### Discussion

Over five decades, several peaks of IR of DHF have been identified in Indonesia. The first clear peak occurred in 1988 (Fig. 1) and our systematic review indicates that this peak was associated with the activities of DENV-3 [33]. However, there was no clear determinant that triggered these high activities of DENV-3 in 1988. Another peak occurred between November 1997 and May 1998, 10 years later, in which DHF outbreaks occurred in 11 provinces [36]. There were also no clear explanation of this outbreak. One potential reason is that massive riots occurred throughout Indonesia in 1998. These riots triggered population movements that may have caused the introduction of certain serotypes or genotypes into naïve populations in various regions of Indonesia. Studies during this time isolated a new genotype within DENV-3 that was never reported in Indonesia prior to 1998 [37, 38]. Our phylogenetic study revealed that these viruses were closely related to viruses that had circulated in Thailand indicating that viruses appear to have been imported into Indonesia, had established local circulation and were associated with an increase in DHF cases.

Since 2000, the IR of DHF increased significantly and peaked in 2009 and 2016 (Fig. 1). There are some plausible explanations of this trend. There was a rapid change of serotype dominancy (serotype shift) in Indonesia in 2000s from DENV-3 to DENV-1 and DENV-2 and there were high activities of multiple serotypes in majority of regions in Indonesia [33]. Interestingly, rapid serotype shifts also occurred in other countries in Southeast Asian in 2000s [39–41] indicating this was a regional phenomenon. These rapid serotype shifts may be the most significant factor contributing to the increasing trend of DHF incidence in Indonesia between 2000 and 2009. There was also an introduction of a new genotype within DENV-1 that was never reported previously. Although this genotype was introduced relative recently, the viruses of this genotype have become the most frequently isolated virus within DENV-1 in Indonesia and have been isolated in almost all Indonesian main islands [15, 20–26, 42–47]. In addition, this trend changed during 2000s in which the predominant serotype was found to be associated with the severe form of dengue infection. For example, in 2009 a study found that patients with DENV-1 were more likely to have severe disease [48]. Interestingly, this study also indicated that all patients with DENV-1, as a primary or secondary infection, had severe clinical manifestations [48]. Other studies also indicated that DENV-1 was more frequently associated with severe dengue infection [15, 20, 25]. Altogether this, in part, explains the increased the IR of DHF in Indonesia during 2000s.

Between 1968 and 1973, there was a sharp reduction of CFR in Indonesia. There are some possible explanations for this. First, this decline was associated with improved management protocols of the diseases in the Community Health Centres or hospitals [16]. Second, this was associated with increased knowledge and awareness both of community members and healthcare providers, and better diagnostics including more sensitive and specific diagnostic tests [16, 27]. All of these factors contributed to development of an improved surveillance system over time, that led to prompt recognition not only of severe cases but also mild cases with low mortality in health care facilities.

In conclusion, over the past five decades, there has been a dramatic increase in IR of DHF in Indonesia with a cyclic pattern that peaked approximately every 6 to 8 years while the annual CFR has decreased, over time.

## Study limitation

The WHO criteria, adopted by MoH of Indonesia, is based on both clinical and diagnostic criteria in which any probable case also should be reported into the surveillance system. The surveillance system did not collect the status of DENV infection (i.e. primary or secondary infection). In addition, this surveillance system does not include mild symptomatic dengue cases and only captured DHF cases presenting to healthcare facilities. Therefore, dengue cases are potentially underreported.

## Data Availability

The datasets generated during and/or analyzed during the current study are available from the corresponding author on reasonable request.

## Abbreviations

CFR: case fatality rate
DENV: dengue virus
DHF: dengue hemorrhagic fever
DSS: dengue shock syndrome
IR: incidence rate
MoH: Ministry of Health
WHO: World Health Organization
